# Supplementary material for: Typical development of the human fetal subplate: regional heterogeneity, growth, and asymmetry assessed by in vivo T2-weighted MRI
Source: bioRxiv. 2025 Oct 7:2025.10.06.680743. Preprint. [Version 1] doi: 10.1101/2025.10.06.680743 (PMC12632292; doi:10.1101/2025.10.06.680743)
Supplement: Supplement 1 [file media-1.pdf]

Supporting Information for

Typical development of the human fetal subplate: regional heterogeneity, growth, and asymmetry assessed by in vivo T2-weighted MRI.

Andrea Gondová*, Jennings Zhang, Sungmin You, Seungyoon Jeong, Milton O. Candela-Leal, Caitlin K Rollins, P. Ellen Gran, Hyuk Jin Yun, Kiho Im*

*Andrea Gondová, andrea.gondova@childrens.harvard.edu
*Kiho Im, kiho.im@childrens.harvard.edu

This PDF file includes:

Supp. Figures 1 to 8
Supp. Tables 1 to 8

Figures

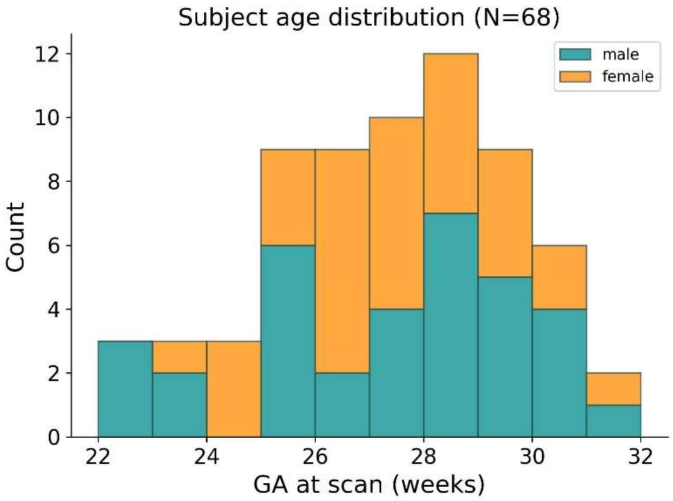

Supp. Figure 1. Age distribution across the cohort.

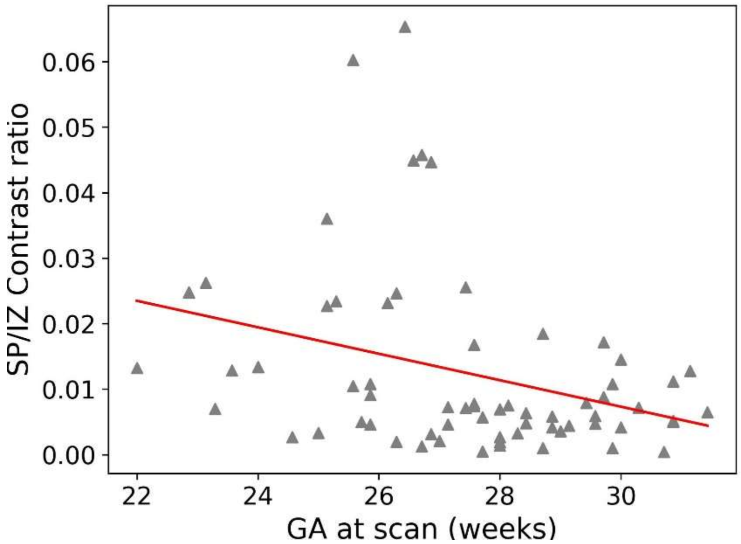

Supp. Figure 2. The contrast ratio of mean intensities at the boundary between SP and IZ (calculated as [mean SP signal - mean IZ signal] / [mean SP signal + mean IZ signal]) decreases with age ($R^2 = -0.334$, $p = 0.005$) making the two regions indistinguishable on T2-weighted MRI scans towards 32wGA.

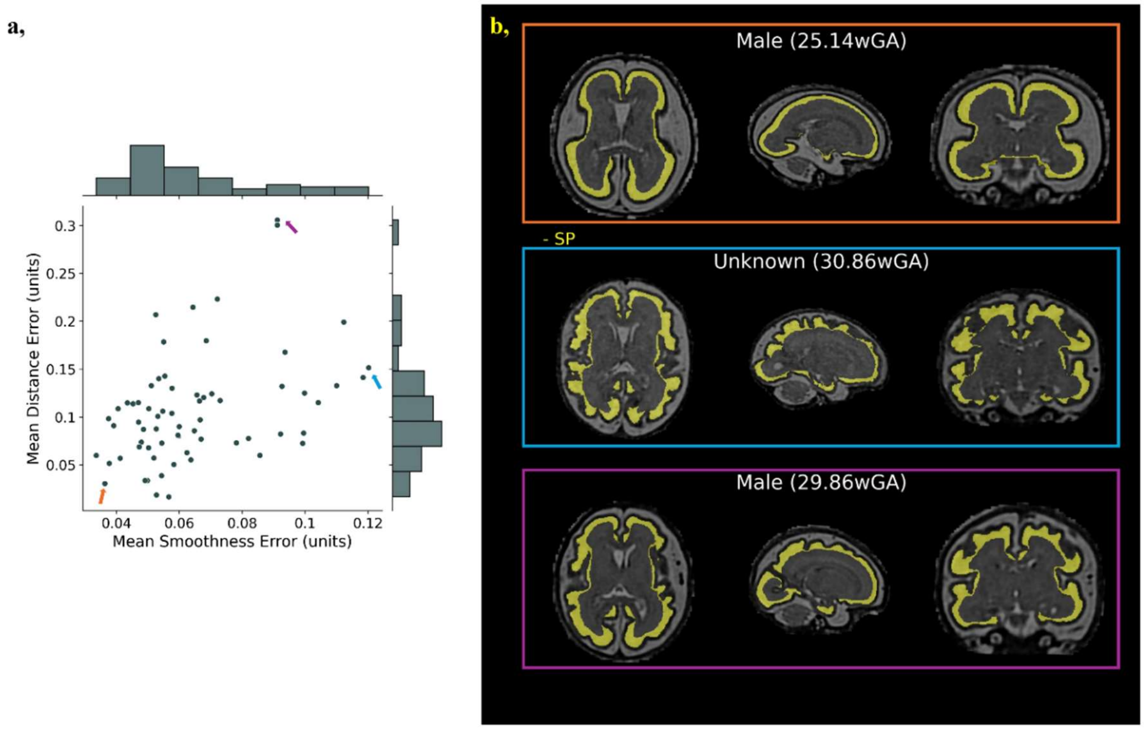

Supp. Figure 3. Surface extraction quality control (outer SP surface) across subjects and **b,** an example T2w reconstruction with overlayed SP segmentation (yellow) in the 31w template space for the subjects with the **best** and worst reconstruction quality (based on **smoothness** and **distance** errors).

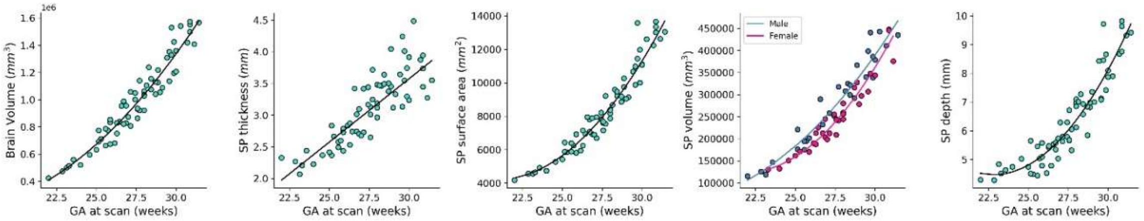

Supp. Figure 4. Relationships between gestational age (GA) and selected brain metrics across the cohort, presented as scatterplots. Each point represents a single subject. Metrics include SP thickness, surface area, thickness, depth, and whole brain volume (for reference). Sex is differentiated where significant sex-related effects or interactions with GA were identified in ANCOVA models (see *Supp. Table 3*): males are shown in blue, females in pink. Lines represent the best-fitting model (linear or quadratic), selected using Akaike Information Criterion (AIC), and are shown only where relationships were statistically significant ($p<0.05$).

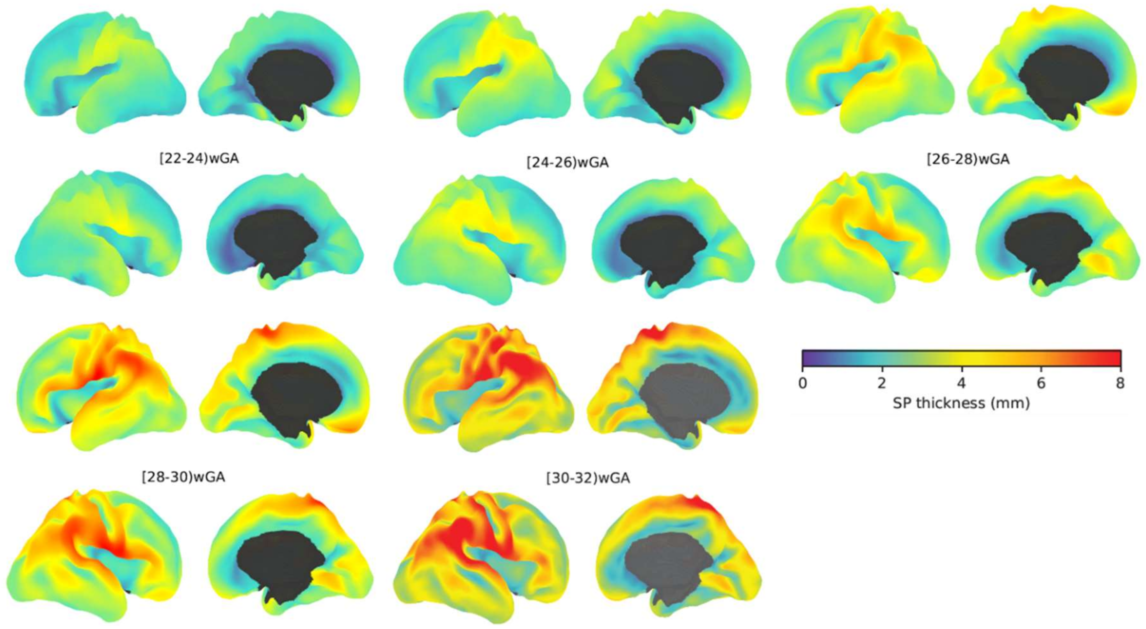

Supp. Figure 5. Example of SP thickness across gestation (22–32 weeks GA). Subjects were grouped into five gestational age bins, and mean vertex-wise SP thickness was computed within each bin for visualization.

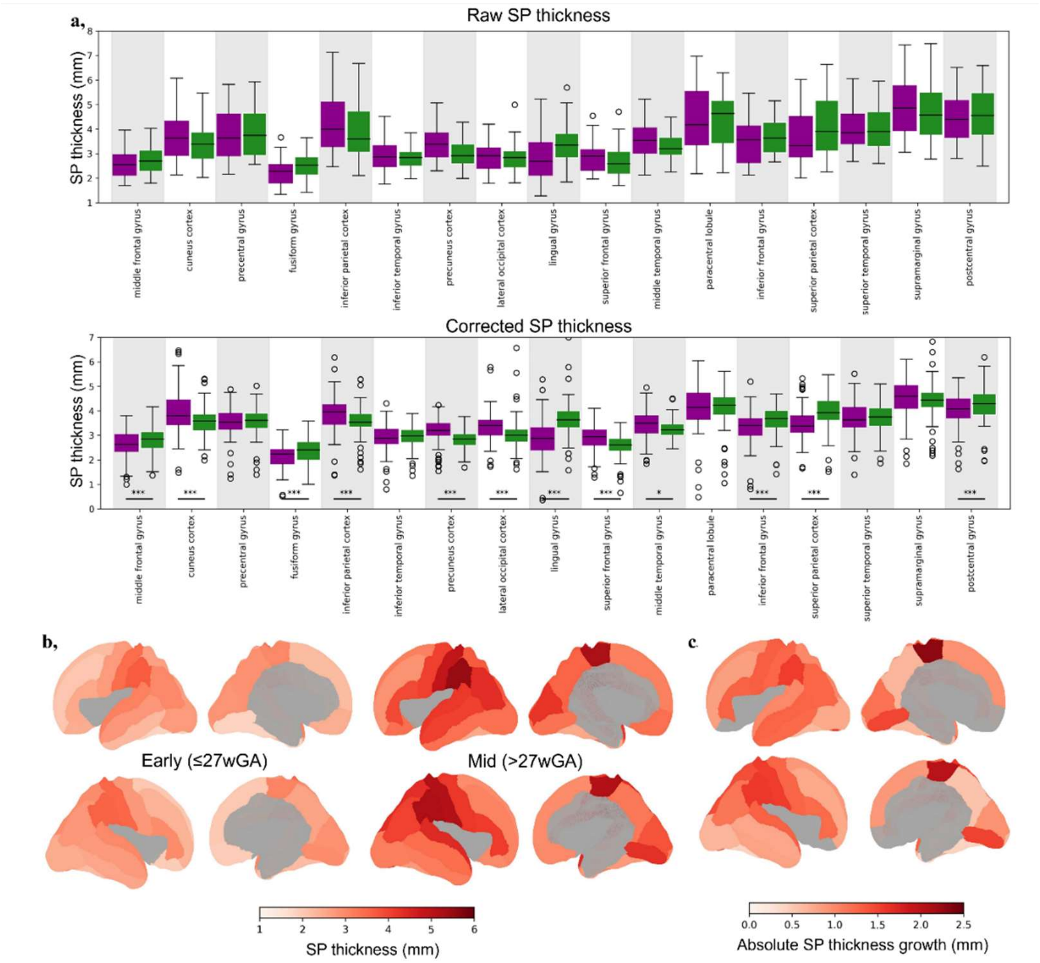

Supp. Figure 6. a, Boxplots summarizing raw regional SP thickness across subjects (top row) and values after correction for GA, residual brain volume, and regional SP sulcal depth (bottom row). After corrections, paired t-tests were used to assess differences between homotopic regions; statistically significant differences after FDR correction are marked with asterisks (* $p < 0.05$, ** $p < 0.01$, *** $p < 0.001$). Full numerical results, including paired comparisons for SP surface area and volume, are provided in *Supp. Table 6*. **b,** Regional SP thickness map comparing early and mid-gestational periods, corrected for covariates. **c,** Map of absolute regional SP thickness growth between early and mid-gestational periods.

a,

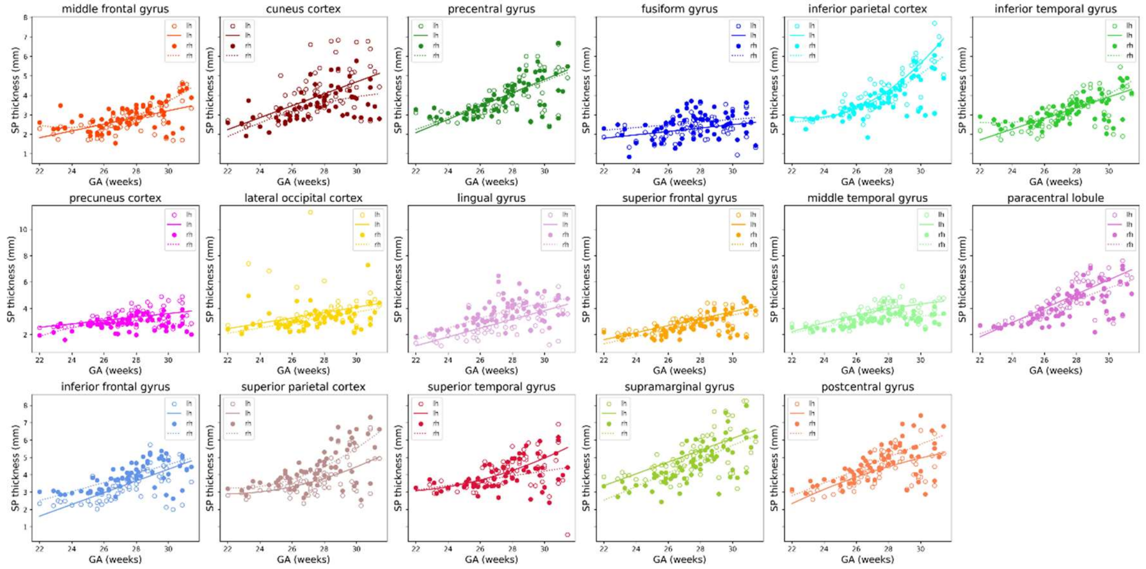

b,

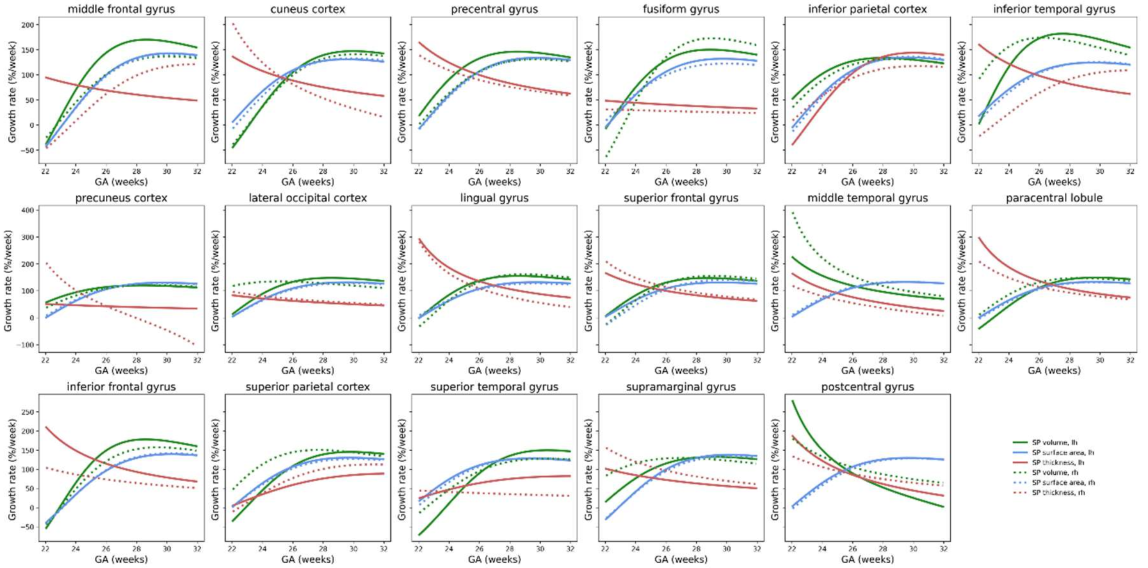

Supp. Figure 7. a, Developmental trajectories of regional SP thickness. Points represent regional thickness values after correction for confounders. The lines/curves show the RANSAC-derived developmental profiles. **b,** Regional relative growth rates (% per week) for SP characteristics sampled across 22–32wGA, based on RANSAC-fitted trajectories and high-resolution temporal sampling (0.1-week intervals). Instantaneous growth rates were computed using finite differences and normalized to account for regional size, yielding a consistent measure of developmental tempo across regions.

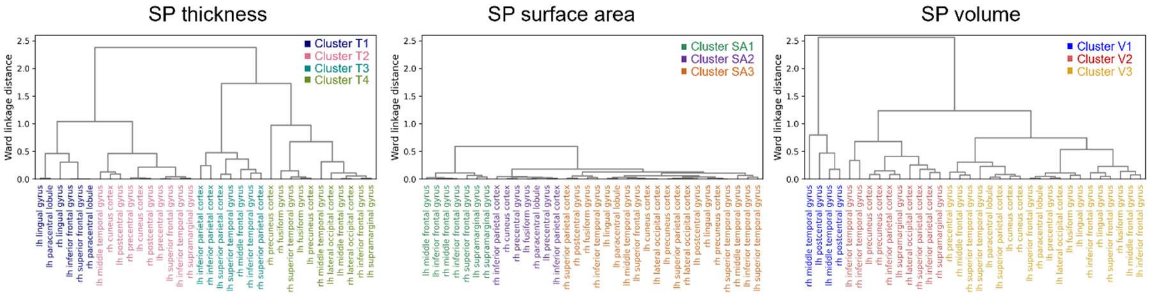

Supp. Figure 8. Dendrogram from hierarchical clustering (Ward's method) based on the developmental profiles shown in *Supp. Figure 7b*. The clustering reveals groups of SP regions with similar developmental trajectories over the studied gestational window.

Tables

Supp. Table 1. Cohort characteristics. Reported p-values compare characteristics between early and mid-gestational periods and result from t-tests for continuous variables and χ² test for sex and maternal variables: categorized maternal BMI and education level.

	TD (N=68)	Early (N=29)	Mid (N=39)	
**Males/Females/Unknown**	34/32/2	13/14/1	21/17/1	χ^2= 0.212, p= 0.645
**GA at scan (weeks) (median [range])**	27.365 [22.00-31.43]	25.57 [22.0-27.0]	28.86 [27.14-31.43]	-
**Maternal age (years) (median [range])**	32.565 [21.79-39.98]	32.92 [22.71-39.90]	32.94 [21.79-39.98]	t=-0.731, p=0.468
**Maternal BMI (median [range])**	26.584, [18.66-44.30] (N=61)	26.58 [19.31-36.76] (N=26)	32.94 [18.66-44.30] (N=35)	t=-0.020, p=0.984
Underweight	0	0	0	χ^2= 0.441, p= 0.802
Healthy Weigh	28	11	14	
Overweight	19	8	10	
Obesity	14	7	5	
**Education**				
< College	1	0	1	χ^2= 1.156, p= 0.764
Partial College/ Specialized training/ High School	14	6	8	
Graduated standard College/University	21	8	13	
Graduate Degree	32	15	17	

Supp. Table 2. 21-label parcellation scheme compared to original DKT cortical regions.

DKT label names	21 label	Refered name
caudal middle frontal gyrus, rostral middle frontal gyrus	1	middle frontal gyrus
cuneus cortex, pericalcarine cortex (upper bank)	2	cuneus cortex
precentral gyrus	3	precentral gyrus
fusiform gyrus	4	fusiform gyrus
inferior parietal lobule	5	inferior parietal cortex
inferior temporal gyrus, temporal pole	6	inferior temporal gyrus
precuneus	7	precuneus cortex
lateral occipical cortex	8	lateral occipical cortex
lingual gurys, pericalcarine cortex (lower bank)	9	lingual gyrus
superior frontal gyrus	10	superior frontal gyrus
middle temporal gyrus, superior temporal sulcus, temporal pole	11	middle temporal gyrus
paracentral lobule	12	paracentral lobule
pars opercularis, pars orbitalis, pars triangularis	13	inferior frontal gyrus
superior parietal cortex	14	superior parietal cortex
superior temporal gyrus, superior temporal sulcus, transverse temporal gyrus, temporal pole	15	superior temporal gyrus
supramarginal gyrus	16	supramarginal gyrus
postcentral gyrus	17	postcentral gyrus
lateral orbitofrontal gyrus, medial orbitofrontal gyrus, frontal pole	18	orbitofrontal gyrus
insula	19	insula
caudal anterior cingulate cortex, posterior cingulate cortex, rostral anterior cingulate cortex, isthmus cingulate cortex	20	cingulate cortex
parahippocampal gyrus, entorhinal cortex	21	parahippocampal cortex

Supp. Table 3. Results of ANCOVA analysis examining the relationship between the SP characteristics and covariates, including GA, residual brain volume (corrected for GA), and sex, along with relevant interactions. Legend: F: F-statistic from ANCOVA; PR (>F): statistical significance; η² = effect size.

		SP thickness			***SP surface area***			***SP volume***			***SP depth***		
		**F**	**PR (>F)**	**η²**	**F**	**PR (>F)**	**η²**	**F**	**PR (>F)**	**η²**	**F**	**PR (>F)**	**η²**
Continuous													
	GA	393.553	<0.001	0.774	6167.471	<0.001	0.950	2823.253	<0.001	0.927	380.265	<0.001	0.850
	residual brain volume	52.507	<0.001	0.103	248.569	<0.001	0.038	156.999	<0.001	0.052	5.542	0.022	0.012
Categorical													
	Sex	1.837	0.180	0.004	1.132	0.292	0.000	5.594	0.021	0.002	0.006	0.939	0.000
Interactions													
	GA : sex	0.263	0.610	0.001	13.120	0.001	0.002	1.059	0.308	0.000	1.650	0.204	0.004
	residual brain volume : sex	0.220	0.641	0.000	0.000	0.993	0.000	0.000	0.988	0.000	0.086	0.770	0.000

Supp. Table 4. Results of ANCOVA examining the relationship between the log-transformed regional SP thickness with SP depth (corrected for GA and residual brain volume), and across regions. The models account for hierarchical structure of the repeated measures across regions and hemispheres with a nested error term. See *Supp. Table 3* for legend.

		SP thickness		
		**F**	**PR (>F)**	**η^2**
Between				
	GA	920.094	<0.001	0.383
	residual brain volume	66.957	<0.001	0.028
	SP depth	20.229	<0.001	0.008
	SP depth : region	2.287	0.014	0.015
Subject:ROI				
	SP depth	190.129	<0.001	0.016
	ROI	282.705	<0.001	0.383
	SP depth : region	2.321	0.002	0.003
Subject:ROI:hemi				
	hemi	3.813	0.051	0.000
	SP depth	295.686	<0.001	0.010
	SP depth : region	6.329	<0.001	0.004

Supp. Table 5. Results of ANCOVA analysis of regional differences and regional hemispheric asymmetries. The models account for hierarchical structure of the repeated measures across regions and hemispheres with a nested error term. See *Supp. Table 3* for legend.

		SP thickness*			***SP surface area***			***SP volume***		
		**F**	**PR (>F)**	**η²**	**F**	**PR (>F)**	**η²**	**F**	**PR (>F)**	**η²**
Between										
	GA	94.698	<0.001	0.260	5519.100	<0.001	0.422	2371.900	<0.001	0.423
	residual brain volume	4.309	0.042	0.012	224.100	<0.001	0.017	141.900	<0.001	0.025
Subject:ROI										
	region	111.956	<0.001	0.271	36981.000	<0.001	0.554	1260.640	<0.001	0.492
	GA : region	9.052	<0.001	0.022	5.550	<0.001	0.000	23.520	<0.001	0.009
Subject:ROI:hemi										
	hemi	8.945	0.003	0.001	70.700	<0.001	0.000	0.744	0.389	0.000
	GA : hemi	2.347	0.126	0.000	13.660	0.001	0.000	2.241	0.135	0.000
	region : hemi	26.278	<0.001	0.025	26.780	<0.001	0.000	29.731	<0.001	0.004
	GA: region : hemi	4.215	<0.001	0.004	3.180	<0.001	0.000	6.808	<0.001	0.001

*SP thickness: residual thickness corrected for regional SP depth within subjects.

Supp. Table 6. Asymmetry analyses. The table reports t-statistics (T) and Cohen's D values derived from one-sample t-tests comparing asymmetry indices to zero to assess significant lateralization. p(corr) denotes FDR-corrected p-values, adjusted for multiple comparisons across regions within each metric.

		SP volume*				***SP surface****				***SP thickness****			
RH-ward	**ROI**	**AI**	**T**	**Cohen's D**	**p (corr.)**	**AI**	**T**	**Cohen's D**	**p (corr.)**	**AI**	**T**	**Cohen's D**	**p (corr.)**
LH-ward	middle frontal gyrus	5.95	5.73	0.61	<0.001	2.23	13.31	0.57	<0.001	5.74	3.19	0.28	<0.001
	cuneus cortex	-8.61	-5.02	0.68	<0.001	-0.52	-2.18	0.15	0.053	-6.36	-3.30	0.39	<0.001
	precentral gyrus	-7.75	-6.72	0.67	<0.001	0.31	2.16	0.11	0.053	1.59	1.11	0.07	0.364
	fusiform gyrus	1.82	2.14	0.16	0.047	0.18	0.89	0.06	0.441	5.86	3.46	0.29	<0.001
	inferior parietal cortex	-5.48	-5.63	0.59	<0.001	0.32	2.00	0.11	0.070	-5.85	-5.10	0.33	<0.001
	inferior temporal gyrus	-0.60	-0.70	0.07	0.588	0.11	0.74	0.04	0.480	2.16	0.49	0.04	0.726
	precuneus cortex	-18.11	-13.02	1.59	<0.001	-1.49	-6.08	0.42	<0.001	-10.57	-6.20	0.74	<0.001
	lateral occipital cortex	2.28	2.30	0.22	0.035	-0.58	-3.39	0.17	0.003	-12.16	-5.39	0.58	<0.001
	lingual gyrus	6.80	4.65	0.60	<0.001	0.32	1.34	0.09	0.244	34.59	11.06	1.31	<0.001
	superior frontal gyrus	-0.01	0.10	0.01	0.950	1.47	8.35	0.48	<0.001	-7.11	-5.51	0.34	<0.001
	middle temporal gyrus	-10.43	-7.38	0.77	<0.001	0.46	2.75	0.14	0.017	-3.95	-2.94	0.30	0.011
	paracentral lobule	13.24	7.15	0.90	<0.001	-0.23	-0.87	0.07	0.441	1.63	-0.01	0.00	0.367
	inferior frontal gyrus	4.00	2.82	0.32	0.010	1.77	10.84	0.43	<0.001	10.48	5.70	0.47	<0.001
	superior parietal cortex	6.35	6.05	0.46	<0.001	0.11	0.71	0.03	0.480	14.22	11.77	0.67	<0.001
	superior temporal gyrus	0.68	0.10	0.01	0.950	0.78	3.43	0.23	0.003	6.51	2.76	0.28	0.353
	supramarginal gyrus	0.00	0.06	0.01	0.950	-0.38	-2.44	0.11	0.033	-1.10	-0.87	0.07	0.229
	postcentral gyrus	7.86	6.10	0.59	<0.001	1.07	7.30	0.37	<0.001	7.02	4.37	0.38	0.001

* SP volume / SP surface: corrected for GA and residual brain volume; SP thickness additionally corrected for regional SP depth within subject.

Supp. Table 7. Differences in relative growth and asymmetry between early and mid-gestational periods. p(corr) denotes FDR-corrected p-values, adjusted for multiple comparisons across regions within each metric. The Left/Right growth columns summarize mean regional values of each SP characteristic at the early and mid-gestational periods. % Growth indicates the relative percent increase over this interval, with corresponding 95% confidence intervals (CI). Left vs. Right columns compare relative percent growth between hemispheres using a Z-test based on the difference in percent change, accounting for propagated standard error. For Asymmetry Indices (AI), values are reported for the early and mid-gestational periods. The adjacent p(corr) columns show FDR-corrected p-values from one-sample t-tests against zero, corrected across regions. ΔAI reflects the absolute change in asymmetry between the two periods, with 95% CI. These changes were also compared using a Z-test that accounts for propagated standard error.

	early ≤ 27wGA, mid > 27wGA			*SP thickness**															
		left growth				right growth				left vs right		AI (%)							
RH-ward	ROI	early	mid	%growth	CI	early	mid	%growth	CI	Z	p (corr.)	early AI	p (corr.)	mid AI	p (corr.)	ΔAI	CI [AI/100]	Z	p (corr.)
LH-ward	middle frontal gyrus	2.15	2.94	36.84	[27.06, 46.62]	2.38	3.14	31.80	[23.07, 40.52]	0.75	0.665	10.57	0.005	6.89	0.000	3.67	[-0.03, 0.11]	1.03	0.537
	cuneus cortex	3.06	4.37	42.88	[30.47, 55.3]	2.92	3.93	34.27	[25.58, 42.97]	1.11	0.646	-3.37	0.396	-8.31	0.037	4.94	[-0.05, 0.15]	1.00	0.537
	precentral gyrus	2.77	4.13	49.00	[39.42, 58.58]	2.87	4.13	44.21	[36.07, 52.34]	0.75	0.665	3.95	0.070	0.30	0.868	3.65	[-0.01, 0.09]	1.40	0.362
	fusiform gyrus	1.88	2.43	28.98	[18.44, 39.53]	2.14	2.70	26.39	[15.08, 37.7]	0.33	0.902	11.29	0.004	11.09	0.000	0.20	[-0.07, 0.08]	0.05	0.959
	inferior parietal cortex	3.12	4.53	45.24	[35.29, 55.18]	2.85	4.17	46.30	[37.33, 55.28]	-0.16	0.964	-8.84	0.003	-7.79	0.000	1.05	[-0.04, 0.06]	0.39	0.845
	inferior temporal gyrus	2.32	3.46	49.26	[39.26, 59.25]	2.54	3.31	30.41	[22.79, 38.03]	2.94	0.056	10.27	0.006	-4.19	0.048	14.47	[0.07, 0.22]	3.93	0.001
	precuneus cortex	2.83	3.55	25.70	[18.08, 33.33]	2.55	3.07	20.40	[12.57, 28.24]	0.95	0.646	-10.92	0.001	-13.88	0.000	2.96	[-0.04, 0.1]	0.80	0.597
	lateral occipital cortex	3.04	3.66	20.45	[2.04, 38.86]	2.62	3.29	25.56	[14.17, 36.95]	-0.46	0.842	-11.70	0.000	-8.60	0.022	3.09	[-0.05, 0.11]	0.74	0.597
	lingual gyrus	2.18	3.39	55.64	[40.71, 70.57]	2.89	4.29	48.73	[37.43, 60.03]	0.72	0.665	29.22	0.000	25.14	0.000	4.09	[-0.06, 0.14]	0.78	0.597
	superior frontal gyrus	2.38	3.26	37.09	[27.97, 46.2]	2.12	3.09	46.10	[36.41, 55.79]	-1.33	0.646	-11.45	0.000	-5.80	0.001	5.66	[0.01, 0.11]	2.24	0.107
	middle temporal gyrus	2.96	3.94	33.16	[24.26, 42.05]	2.84	3.46	21.86	[14.77, 28.94]	1.95	0.437	-3.40	0.151	-12.45	0.000	9.05	[0.03, 0.15]	2.95	0.027
	paracentral lobule	3.12	5.04	61.81	[50.2, 73.43]	3.12	5.04	61.46	[51.14, 71.78]	0.05	0.964	0.53	0.819	1.13	0.663	0.59	[-0.05, 0.06]	0.21	0.948
	inferior frontal gyrus	2.68	3.88	44.62	[34.83, 54.41]	3.06	4.16	35.93	[28.41, 43.45]	1.38	0.646	13.85	0.000	7.68	0.001	6.17	[0, 0.12]	2.03	0.143
	superior parietal cortex	2.74	3.97	44.98	[34.98, 54.98]	3.08	4.69	52.08	[42.5, 61.67]	-1.00	0.646	11.53	0.000	17.05	0.000	5.52	[0.01, 0.1]	2.41	0.090
	superior temporal gyrus	3.22	4.10	27.54	[17.72, 37.36]	3.14	4.21	34.11	[26.24, 41.98]	-1.02	0.646	-2.45	0.352	5.21	0.365	7.66	[-0.03, 0.19]	1.37	0.362
	supramarginal gyrus	3.72	5.39	45.12	[36.67, 53.58]	3.57	5.18	44.80	[36, 53.59]	0.05	0.964	-4.24	0.070	-4.01	0.100	0.23	[-0.06, 0.06]	0.08	0.959
	postcentral gyrus	3.46	4.70	35.91	[27.72, 44.1]	3.48	4.97	42.86	[33.62, 52.1]	-1.10	0.646	-0.01	0.996	5.13	0.037	5.14	[-0.01, 0.11]	1.71	0.247

	*SP surface**																	
	left growth				right growth				left vs right		AI (%)							
ROI	early	mid	%growth	CI	early	mid	%growth	CI	Z	p (corr.)	early AI	p (corr.)	mid AI	p (corr.)	ΔAI	CI [AI/100]	Z	p (corr.)
middle frontal gyrus	235.42	385.16	63.61	[62.25, 64.96]	235.42	393.80	67.28	[61.93, 65.04]	0.12	0.962	2.28	0.000	2.20	0.000	0.08	[-0.005, 0.007]	0.26	0.842
cuneus cortex	91.67	151.51	65.29	[63.92, 66.65]	91.67	150.87	64.59	[64.28, 67.28]	-0.47	0.960	-0.73	0.015	-0.43	0.263	0.31	[-0.005, 0.011]	0.76	0.786
precentral gyrus	195.97	321.92	64.27	[63.17, 65.37]	195.97	322.80	64.72	[63.07, 65.17]	0.19	0.960	0.37	0.039	0.28	0.185	0.09	[-0.004, 0.005]	0.39	0.786
fusiform gyrus	106.05	174.17	64.22	[62.88, 65.57]	106.05	174.68	64.71	[63.51, 66.17]	-0.64	0.960	-0.08	0.721	0.30	0.311	0.38	[-0.003, 0.01]	1.17	0.688
inferior parietal cortex	157.19	257.21	63.63	[62.43, 64.83]	157.19	257.86	64.05	[62.15, 64.46]	0.39	0.960	0.46	0.079	0.25	0.263	0.21	[-0.004, 0.008]	0.71	0.786
inferior temporal gyrus	108.44	176.73	62.98	[61.82, 64.13]	108.44	177.38	63.57	[63.04, 65.51]	-1.50	0.960	-0.43	0.024	0.36	0.155	0.79	[0.003, 0.013]	3.05	0.023
precuneus cortex	153.11	251.21	64.07	[62.74, 65.4]	153.11	247.79	61.84	[63.24, 66.17]	-0.63	0.960	-1.77	0.000	-1.37	0.000	0.40	[-0.004, 0.012]	0.96	0.786
lateral occipital cortex	119.09	195.19	63.90	[62.6, 65.2]	119.09	193.96	62.87	[62.33, 64.96]	0.27	0.960	-0.48	0.009	-0.63	0.014	0.15	[-0.004, 0.007]	0.56	0.786
lingual gyrus	125.33	207.24	65.36	[63.83, 66.9]	125.33	208.00	65.97	[64.19, 67.15]	-0.28	0.960	0.18	0.440	0.37	0.268	0.19	[-0.005, 0.009]	0.53	0.786
superior frontal gyrus	266.60	440.84	65.35	[64.13, 66.58]	266.60	446.59	67.51	[63.43, 65.59]	1.02	0.960	1.81	0.000	1.30	0.000	0.51	[-0.001, 0.011]	1.77	0.327
middle temporal gyrus	121.32	197.96	63.17	[61.87, 64.47]	121.32	199.32	64.29	[63.02, 65.64]	-1.23	0.960	-0.02	0.920	0.68	0.009	0.70	[0.001, 0.013]	2.30	0.122
paracentral lobule	60.42	99.51	64.70	[63.24, 66.16]	60.42	99.45	64.60	[64.15, 67.13]	-0.88	0.960	-0.63	0.015	-0.06	0.877	0.58	[-0.002, 0.014]	1.38	0.565
inferior frontal gyrus	122.17	200.92	64.46	[63.04, 65.87]	122.17	204.46	67.36	[62.64, 65.86]	0.19	0.960	1.86	0.000	1.73	0.000	0.13	[-0.004, 0.007]	0.45	0.786
superior parietal cortex	154.49	254.36	64.65	[63.41, 65.89]	154.49	254.63	64.83	[63.37, 65.96]	-0.02	0.982	0.09	0.721	0.11	0.591	0.02	[-0.005, 0.005]	0.06	0.950
superior temporal gyrus	165.20	271.39	64.28	[62.85, 65.71]	165.20	273.72	65.68	[63.43, 66.01]	-0.46	0.960	0.59	0.024	0.86	0.013	0.27	[-0.004, 0.01]	0.74	0.786
supramarginal gyrus	120.39	197.91	64.39	[63.16, 65.62]	120.39	196.64	63.33	[61.69, 64.27]	1.55	0.960	0.21	0.440	-0.64	0.004	0.85	[0.003, 0.014]	3.00	0.023
postcentral gyrus	168.12	276.14	64.25	[63.12, 65.38]	168.12	279.00	65.96	[63.03, 65.14]	0.21	0.960	1.14	0.000	1.04	0.000	0.10	[-0.004, 0.006]	0.40	0.786

SP volume*

ROI	left growth				right growth				left vs right		AI (%)							
	early	mid	%growth	CI	early	mid	%growth	CI	Z	p (corr.)	early AI	p (corr.)	mid AI	p (corr.)	ΔAI	CI [AI/100]	Z	p (corr.)
middle frontal gyrus	6698.81	13415.86	100.27	[95.33, 105.22]	7186.82	14228.06	97.97	[95.33, 103.49]	0.61	0.577	6.81	0.002	5.82	0.000	0.99	[-0.03, 0.05]	0.46	0.683
cuneus cortex	3148.51	5404.52	71.65	[66.46, 76.85]	3019.52	5105.18	69.07	[66.46, 74.53]	0.67	0.569	-4.16	0.147	-5.78	0.014	1.61	[-0.04, 0.08]	0.54	0.671
precentral gyrus	6940.22	12702.99	83.03	[78.45, 87.62]	6919.08	11438.01	65.31	[78.45, 69.83]	5.40	0.000	-0.32	0.934	-10.44	0.000	10.12	[0.06, 0.14]	4.91	0.000
fusiform gyrus	2396.05	4768.00	98.99	[92.74, 105.25]	2411.88	4911.63	103.64	[92.74, 110.34]	-0.99	0.418	0.38	0.934	2.87	0.071	2.49	[-0.03, 0.08]	0.89	0.485
inferior parietal cortex	6717.28	12385.97	84.39	[79.85, 88.93]	6038.96	11806.62	95.51	[79.85, 100.44]	-3.25	0.004	-10.87	0.000	-4.80	0.000	6.07	[0.01, 0.11]	2.40	0.035
inferior temporal gyrus	3327.79	6882.13	106.81	[101.73, 111.89]	3382.60	6748.54	99.51	[101.73, 104.12]	2.09	0.063	1.81	0.644	-1.95	0.070	3.76	[-0.01, 0.09]	1.48	0.213
precuneus cortex	4890.59	8263.66	68.97	[64.12, 73.83]	4198.53	6710.08	59.82	[64.12, 64.28]	2.72	0.016	-15.29	0.000	-20.41	0.000	5.12	[0.01, 0.09]	2.46	0.035
lateral occipital cortex	3954.77	7307.88	84.79	[80.36, 89.22]	3970.11	7847.78	97.67	[80.36, 102.68]	-3.77	0.001	0.27	0.934	7.00	0.000	6.73	[0.02, 0.11]	3.00	0.013
lingual gyrus	3045.14	5663.32	85.98	[81.57, 90.39]	3175.21	6226.24	96.09	[81.57, 100.86]	-3.05	0.007	4.21	0.005	9.30	0.000	5.08	[0.01, 0.09]	2.35	0.036
superior frontal gyrus	8057.76	15153.82	88.06	[84.56, 91.57]	7740.81	15646.58	102.13	[84.56, 106.52]	-4.91	0.000	-4.18	0.043	3.13	0.003	7.31	[0.04, 0.11]	3.91	0.001
middle temporal gyrus	4682.51	7813.79	66.87	[61.11, 72.64]	4278.97	6955.96	62.56	[61.11, 67.56]	1.11	0.380	-8.65	0.000	-11.80	0.000	3.14	[-0.02, 0.08]	1.28	0.283
paracentral lobule	1853.78	3298.27	77.92	[71.62, 84.23]	1896.76	3669.81	93.48	[71.62, 98.55]	-3.77	0.001	2.50	0.178	10.94	0.000	8.43	[0.03, 0.14]	2.94	0.013
inferior frontal gyrus	3423.60	6535.41	90.89	[84.99, 96.8]	3675.06	6696.62	82.22	[84.99, 86.8]	2.27	0.043	7.26	0.011	2.71	0.099	4.55	[-0.01, 0.1]	1.66	0.165
superior parietal cortex	5467.88	9786.54	78.98	[73.12, 84.84]	5603.51	10599.01	89.15	[73.12, 94.45]	-2.52	0.025	2.50	0.184	8.14	0.000	5.64	[0.02, 0.09]	2.89	0.013
superior temporal gyrus	5289.55	8421.89	59.22	[52.12, 66.31]	5254.66	8462.13	61.04	[52.12, 66.26]	-0.41	0.685	-0.38	0.934	1.32	0.583	1.70	[-0.04, 0.07]	0.61	0.657
supramarginal gyrus	4712.92	8383.54	77.88	[72.97, 82.8]	4737.76	8283.83	74.85	[72.97, 81.04]	0.75	0.548	-0.02	0.993	-1.30	0.570	1.28	[-0.05, 0.08]	0.40	0.688
postcentral gyrus	6537.66	9146.77	39.91	[34.27, 45.55]	6683.09	9906.64	48.23	[34.27, 54.13]	-2.00	0.070	2.17	0.236	7.99	0.000	5.82	[0.01, 0.11]	2.42	0.035

Supp. Table 8. Silhouette scores quantifying the quality of different clustering solutions. Higher scores indicate better separation and cohesion within clusters.

	Silhouette score		
**Cluster number**	**SP volume**	**SP surface area**	**SP thickness**
2	0.655	0.709	0.465
3	0.361	0.406	0.507
4	0.357	0.423	0.513
5	0.331	0.408	0.575
6	0.356	0.438	0.603
7	0.357	0.430	0.559
8	0.350	0.419	0.556
9	0.358	0.368	0.514
10	0.360	0.385	0.463
11	0.343	0.367	0.451
12	0.328	0.347	0.460
13	0.327	0.352	0.451
14	0.319	0.354	0.467
15	0.288	0.342	0.506
16	0.280	0.354	0.481
17	0.263	0.335	0.453
18	0.260	0.367	0.457
19	0.241	0.322	0.443
20	0.229	0.314	0.459
21	0.209	0.291	0.446
22	0.194	0.298	0.453
23	0.190	0.284	0.461
24	0.182	0.266	0.410
25	0.169	0.262	0.380
26	0.147	0.246	0.365
27	0.132	0.216	0.316
28	0.118	0.202	0.280
29	0.107	0.172	0.236
30	0.085	0.130	0.197
31	0.067	0.107	0.157
32	0.048	0.098	0.113
33	0.048	0.098	0.113

optimal number (highest Silhouette score)

final cluster number
